# Roles of Bone-Marrow-Derived Cells and Inflammatory Cytokines in Neointimal Hyperplasia after Vascular Injury

**DOI:** 10.1155/2014/945127

**Published:** 2014-01-14

**Authors:** Makoto Shoji, Shinji Koba, Youichi Kobayashi

**Affiliations:** Department of Medicine, Division of Cardiology, Showa University School of Medicine, 1-5-8 Hatanodai, Shinagawa-ku, Tokyo 142-8555, Japan

## Abstract

Bone-marrow-derived cells can generate vascular progenitor cells that contribute to pathological remodeling in models of restenosis after percutaneous coronary intervention (PCI). We created models of vascular injury in mice with bone marrow transplants (BMT) to determine relationships between bone-marrow-derived cells and subsequent biological factors. Mesenchymal stromal cells (MSCs) seemed to inhibit the inflammatory reaction and help stabilize injured vascular regions through mobilizing more endogenous bone-marrow-derived (EBMD) cells to the peripheral circulation. Granulocyte-colony stimulating factor (G-CSF) mobilized more EBMD cells to the peripheral circulation, and they accumulated on the injured side of the vascular lumen. The inflammatory cytokines, tumor necrosis factor (TNF)-alpha, and interleukin (IL)-6 mobilized EBMD cells that play an important role in the process of neointimal hyperplasia after vascular injury. These factors might comprise a mechanism that alters the transdifferentiation or paracrine capabilities of EBMD cells and are potential targets of treatment for patients with cardiovascular diseases.

## 1. Introduction

The mechanisms of neointimal hyperplasia should be clarified to treat restenosis after percutaneous coronary intervention (PCI). Smooth muscle cells (SMCs) in the neointima are derived from the media of injured arteries [1, 2]. On the other hand, bone-marrow-derived cells might generate progenitors and potentially contribute to vascular remodeling [3, 4]. Moreover, among the many factors related to the mobilization of bone-marrow-derived cells, local inflammation by cytokines might drive these cells to the vascular wall, resulting in neointimal hyperplasia [5–7].

The relationship between bone-marrow-derived cells and biological markers and how these cells participate in neointimal formation remain unknown.

Mesenchymal stromal cells (MSCs) migrate to engraft into injured tissues where they secrete a large number of cytokines. However, details of the mechanism of action remain obscure.

The effects of granulocyte-colony stimulating factor (G-CSF) on bone marrow cells in relation to vascular lesions have not yet been fully clarified, especially during the process of neointimal hyperplasia. The inflammatory cytokines, tumor necrosis factor (TNF)-*α*, and interleukin (IL)-6 are associated with atherosclerosis, as they induce intercellular adhesion molecule-1 (ICAM-1), which leads to the accumulation of leukocytes and inflammatory cells in vessel lumina and the modulation of SMC proliferation. However, the relationship between the inflammatory reactions of TNF-*α* or IL-6 and bone marrow cell invasion awaits clarification.

Here, we investigated the relationship between bone-marrow-derived cells and these inflammatory cytokines during the development of neointimal hyperplasia in mouse models of vascular injury. This review describes the pathological mechanisms involved in neointimal hyperplasia after vascular injury with the aim of developing strategies for treating restenosis after PCI or atherosclerosis.

## 2. Bone-Marrow-Derived Cells

The mechanism through which bone-marrow-derived cells participate in neointimal formation remains obscure. Here, we extensively characterized the cellular components of neointimal hyperplasia after mechanical vascular injury. We also examined whether bone-marrow-derived cells differentiate into SMC-like cells in vitro and in vivo.

**Figure 1:**
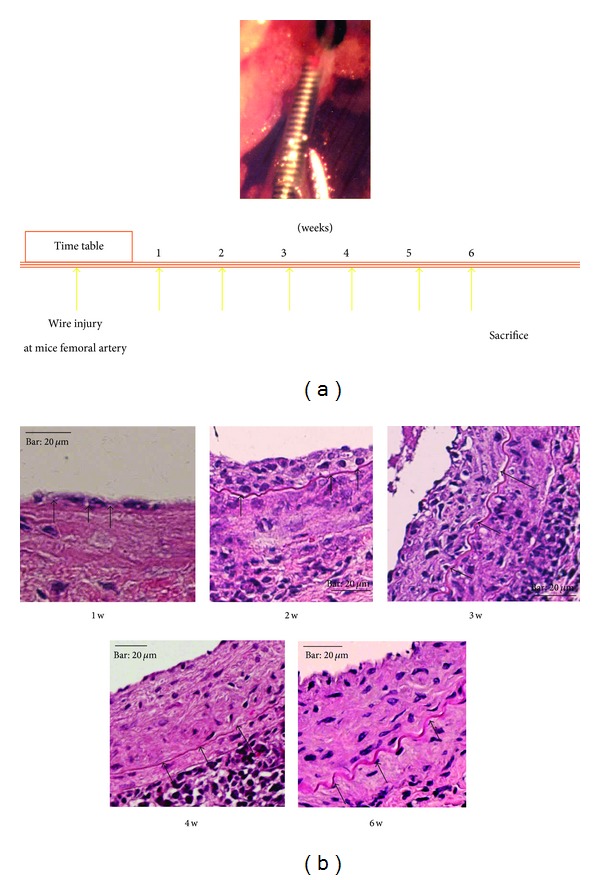
Development of neointima in injured mouse arteries. Femoral arteries of adult male wild-type mice were injured by inserting large wires. Blood flow was restored and injured arteries were harvested at indicated time points. Small neointima is evident on luminal side of injured artery at one week after injury. Large neointima has grown on luminal side at three weeks after injury. Neointima and media at six weeks after injury contain very few CD45^+^ cells and neointima is predominantly composed of cells positive for *α*-SM actin. Arrowheads, internal elastic lamina. Bar, 20 *μ*m.

A large wire was inserted into the femoral artery of adult male wild-type (WT) mice. Thereafter, blood flow was restored and the injured arteries were harvested at the indicated time points (Figure 1). The wire completely denuded the endothelium and obviously enlarged the lumen with the acute onset of medial SMC apoptosis. Cross-sections were stained for *α*-SM actin or CD45 using the avidin-biotin complex. The cellular components of the injured arterial wall were examined by RT-PCR using RNA obtained from total homogenates of vessel walls. Total bone marrow cells were harvested and cultured in the presence of platelet-derived growth factor-BB (PDGF-BB) for a study in vitro.

The expression of *α*-SM actin was obviously downregulated one week after injury but gradually increased after two weeks and reached the level of the undamaged artery at four and six weeks after injury (Figure 1). The cellular components of the arterial wall were immunohistochemically evaluated. Small neointima formed on the luminal side of the injured artery one week after injury. We then assessed the expression of CD45, which indicates cells of hematopoietic lineage derived from bone marrow, in the neointima to determine the involvement of these cells in the peripheral blood after vascular injury. Most neointimal cells expressed CD45, but not *α*-SM actin. Large neointima had formed on the luminal side at three weeks after injury. A few cells were CD45^+^, particularly in the luminal side of the neointima, and some cells were positive for *α*-SM actin. Few cells expressed CD45 in the neointima or media at six weeks after injury, and the neointima predominantly comprised cells that expressed *α*-SM actin. Transmission and scanning electron microscopy showed that endothelium lined the luminal side of the internal elastic lamina of uninjured arteries. The artery remained dilated with a thin media containing only a few cells at two hours after injury, when fibrin was deposited and platelets had accumulated on the denuded luminal side. Direct evidence that bone marrow differentiates into smooth muscle cells was found in vitro. We seeded bone marrow cells on plastic dishes in medium containing serum, removed floating cells four days thereafter, and added PDGF-BB to the adherent cells. Immunocytochemistry and RT-PCR confirmed that some adherent cells differentiated into smooth muscle-like cells that expressed *α*-SM actin under these conditions, unlike the bone marrow cells before culture. We then created models of vascular injury by transplanting wild mice with bone marrow cells from GFP or ROSA26 mice. We identified GFP or LacZ positive cells in neointima after vascular injury. Immunofluorescence staining showed that some of these cells were positive for both smooth muscle and endothelial markers [8].

These findings indicated that endogenous bone-marrow-derived (EBMD) cells contribute to neointimal formation and transdifferentiate into immature SMCs or endothelium at injured vascular sites. Endothelial injury and the subsequent deposition of platelets and fibrin might provide optimal conditions for the homing and differentiation of circulating smooth muscle progenitors. The accumulation of immature smooth muscle cells in the neointimal portion would increase the intima-media ratio. Bone marrow might be an additional source of vascular progenitor cells that can contribute to the repair and remodeling of vessel walls.

## 3. Mesenchymal Stromal Cells from Bone Marrow

Mesenchymal stromal cells (MSCs) are typically defined as adherent, fibroblastoid-like cells that differentiated into osteoblasts, adipocytes, and chondrocytes in vitro [9]. Mesenchymal stromal cells express the surface receptors CD29, CD44, CD49a–f, CD51, CD73, CD105, CD106, CD166, and Stro1 but not the definitive hematopoietic linage markers CD11b, CD14 and CD45 [10]. Mesenchymal stromal cells localize along the endosteal surface of bone marrow and also in a vascular associated niche [11]. These cells play a critical role in regulating the proliferation, differentiation, and quiescence of hematopoietic stem cells in vivo by signalling via the “stem cell niche synapse” through which growth factors, cytokines, and immunomodulatory factors are exchanged [12, 13]. In addition to regulating hematopoietic cells, some nonhematopoietic progenitors might enter the bloodstream and circulate as “continuous reservoirs” of replacement or reparative cells for nonhematopoietic tissues [14]. Because they are pluripotent and easily isolated and expanded, MSCs are of interest as potential therapeutic agents [15, 16]. These cells can migrate to engraft into injured tissues and secrete large numbers of cytokines [17, 18]; however, details of the mechanisms of action remain obscure.

We created models of restricted vascular flow by permanently ligating mouse carotid arteries and injecting the mice with human MSCs (hMSCs) to determine their effects on vascular lesions.

**Figure 2:**
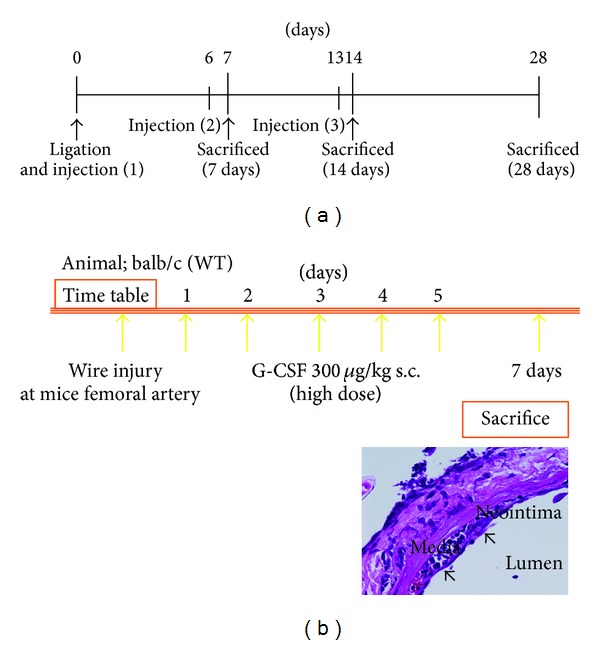
Injury protocols for creating model mice. Time course of carotid artery ligation model (a). Immediately and at six and 13 days after carotid ligation, the left cardiac ventricles of mice were injected with either HBSS (150 *μ*L) or hMSCs (1 × 10^6^ in 150 *μ*L of HBSS) and the mice were sacrificed at 7, 14, and 28 days. Neointimal hyperplasia continued to increase for up to 28 days after ligation. Protocol for G-CSF administration model (b). Mice received daily subcutaneous injections of saline or G-CSF (300 *μ*g/kg) for five days after vascular injury. Neointimal proliferation was induced by G-CSF through early excessive inflammation and bone marrow cell mobilization.

Flow-restriction vascular injury was caused by ligating the bifurcation of the left common carotid artery in mice. Immediately after ligation as well as at six and 13 days thereafter, either HBSS (150 *μ*L) or hMSCs (1 × 10^6^ in 150 *μ*L of HBSS) were injected into the left cardiac ventricle and the mice were sacrificed at 7, 14, and 28 days thereafter (Figure 2(a)). The carotid arteries were carefully excised and assayed using histology or PCR.

**Figure 3:**
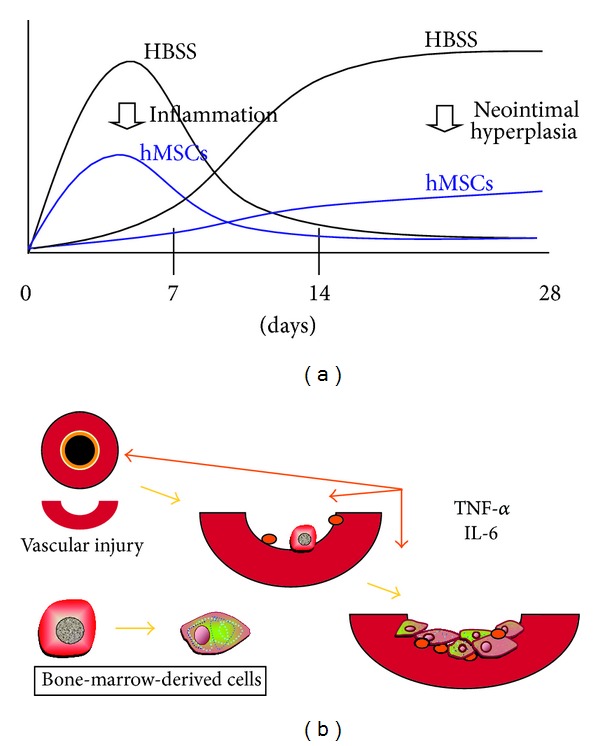
Schematic illustrations of effects of hMSCs on ligated carotid artery (a) and inflammatory cytokines on injured artery (b). Systemic administration of hMSCs decreased neointimal hyperplasia in mouse model without significant long-term engraftment into lesion (a). Rather, modulation of inflammatory response conferred therapeutic benefits. Inflammatory cytokines signal a need for repair and mobilized endogenous bone marrow cells play an important role in neointimal hyperplasia development after vascular injury (b). Inflammatory cytokines might contribute to activation, migration, and proliferation of smooth muscle progenitor cells at sites of injury, through mobilization of bone-marrow-derived cells.

The amount of neointimal hyperplasia was significantly reduced after injecting the hMSCs. Macrophage infiltration at ligated arteries and serum levels of chemoattractive protein-1 (MCP-1) were decreased. However, PCR assays did not detect hMSCs in the carotid arteries. Sections of arteries assayed by immunohistochemistry using a human specific antinuclear antibody did not detect human cells in the mouse carotid arteries [19]. These findings showed that the systemic administration of hMSCs decreased neointimal hyperplasia in a mouse model without significant long-term engraftment into the lesion. Rather, the therapeutic benefit of the cells was probably brought about via the modulation of inflammatory responses (Figure 3(a)).

We assessed serum levels of MCP-1 as a potential biomarker for the therapeutic effects of hMSCs in mice fed with a high-fat (HF) diet, which is an established model of ubiquitous atherosclerotic change, to clarify the role of MCP-1 and hMSCs in atherosclerosis.

Human MSCs were infused into the left cardiac ventricles of mice fed a HF diet, and MCP-1 levels in serum were assayed using ELISA to evaluate changes in inflammatory responses. Baseline levels of MCP-1 in mouse serum at 24 h before injection did not differ between each group. Levels of MCP-1 were higher in mice injected with PBS than with hMSCs 24 h before being fed the HF diet for 12 weeks. Our results indicated that serum MCP-1 levels in the mouse model of atherosclerosis were significantly decreased after hMSC administration [19]. Because MCP-1 has direct proatherogenic properties in addition to its ability to amplify the inflammatory cascade, it might have played a major role in inhibiting neointimal hyperplasia after surgery in this experimental model.

The inhibition of inflammatory reactions via the paracrine effects of exogenous hMSCs might have helped to stabilize the injured vascular regions. Moreover, MSCs might mobilize more endogenous bone-marrow-derived cells to the peripheral circulation, and then the cells could accumulate at injured sites where they would modulate the inflammatory response.

## 4. Inflammatory Cytokines

### 4.1. Granulocyte-Colony Stimulating Factor

Granulocyte-colony stimulating factor (G-CSF) has pleiotropic actions, including cytoprotective and antiapoptotic effects [20]. This factor also improves cardiac function after acute myocardial infarction by mobilizing bone-marrow-derived cells, most prominent endothelial progenitor cells (EPCs), into the peripheral blood [21–27]. Endothelial progenitor cells are generally defined as those that express hematopoietic stem cell markers such as CD34, CD133, and VEGFR2. Both EPCs and hematopoietic stem cells share many surface marker antigens and are descended from precursor hemangioblasts during embryonic development [28, 29].

However, the effects of G-CSF on bone marrow cells in relation to vascular injury have not been fully clarified, especially during the development of neointimal hyperplasia. We administered human recombinant G-CSF to mice with vascular injuries to determine whether G-CSF affects luminal narrowing by promoting neointimal hyperplasia or by accelerating reendothelialization.

A large wire was inserted into the femoral artery of adult male WT mice and then the mice were injected subcutaneously with saline or with G-CSF (300 *μ*g/kg) daily for five days thereafter (Figure 2(b)). We then evaluated neointimal hyperplasia and reendothelialization.

CD34^−^/Sca-1^+^ cells indicating hematopoietic stem cells were more abundant in the G-CSF than the control group at three days after injury according to FACS analysis. The two groups did not significantly differ at 28 days after vascular injury. We found that G-CSF induced neointimal proliferation through early excessive inflammation and bone marrow cell mobilization (Figure 2(b)). However, it subsequently induced early reendothelialization and thus inhibited neointimal hyperplasia [30]. Immunohistological findings from the early phase showed that neointimal hyperplasia comprised many CD34^+^ cells, suggesting that the stem cells mobilized by G-CSF affected the process of neointimal hyperplasia and elevated the intima-media ratio. After recruiting inflammatory cells, mobilized EBMD cells at the later phase tended to transform into EPCs, which might promote endothelialization.

Bone marrow cells from mice were cultured in vitro with G-CSF (1 or 10 *μ*g/mL). At day 14, 1A4^+^ smooth muscle cells were significantly more abundant among the bone marrow cells cultured with, than without, 1 or 10 *μ*g/mL of G-CSF [30]. We found that G-CSF modulated vascular repair through the mobilization of progenitor cells from bone marrow into the peripheral blood. This factor might induce neointimal vulnerability by mobilizing smooth muscle progenitor cells during the early phase of vascular repair and it also accelerates reendothelialization, possibly via the mobilization of endothelial progenitor cells and the promotion of vascular healing after injury during the later phase. Our studies in vitro also showed that G-CSF can modulate the differentiation of mouse bone marrow cells into SM-like cells. In fact, G-CSF mobilized more bone marrow stem cells to the peripheral circulation, where they accumulated on the injured side of the vascular lumina. We also found that some of the cells could transdifferentiate into SM-like cells, as we immunohistochemically identified cells that were double positive for CD34 and immature SMC antibody in the neointimal portion. In contrast to the early phase, the intima-media ratio was smaller in G-CSF than the control group at the later phase. Thus, G-CSF might promote reendothelialization by mobilizing EPCs after the apoptotic cells have been eliminated and neointimal hyperplasia has matured. The progress of atherosclerosis can be prevented by G-CSF at optimal doses. 

### 4.2. TNF-*α* and IL-6

Among the many factors associated with the mobilization of bone marrow cells, local inflammation induced by cytokines might drive bone marrow cells to the vascular wall. The inflammatory cytokine tumor necrosis factor (TNF)-*α* is mainly produced by activated monocytes and macrophages that elicit cytotoxic activity and the activation of various types of cells via signal transduction. Neointimal hyperplasia induced by low shear stress was modulated by TNF-*α*. Interleukin (IL)-6 is associated with atherosclerosis, as it induces intercellular adhesion molecule-1 (ICAM-1), which leads to the accumulation of leukocytes and inflammatory cells in vessel lumina and modulates SMC proliferation [5, 31–35]. However, the relationship between the inflammatory reactions of TNF-*α* or IL-6 and bone marrow cell invasion has not been fully clarified.

Here we injured the arteries of TNF-*α* KO mice to determine the involvement of inflammatory cytokines in the mobilization of EBMD cells during neointimal formation.

The femoral arteries of WT or TNF-*α* KO mice were injured with wire as described above and then sacrificed four weeks later for morphometric analysis of the arteries.

The neointimal area was smaller and fewer inflammatory cells such as neutrophils, macrophages, and apoptotic cells were evident in the neointima of KO than WT mice. Furthermore, reendothelialization appeared earlier in KO than WT.

Immunocytochemical assessment showed that WT and TNF-*α* KO mouse bone marrow cells cultured in vitro differentiated into SM-like cells expressing *α*-SM actin, which they did not express before culture. Fewer cells were positive for *α*-SM actin in the KO than in the WT group. The intima-media ratios were significantly lower at four weeks after vascular injury in the TNF-*α* KO model mice. Apoptosis and inflammatory reactions were evident in the vascular wall after injury in both WT and TNF-alpha KO mice, but fewer apoptotic and inflammatory cells were detected in TNF-*α* KO mice. Furthermore, more cells were CD34^+^ in areas containing more inflammatory cells in WT than KO mice.

Ozkok et al. indicated that TNF-*α* might have specific inhibitory actions against EPC [36]. Thus, inflammatory cell invasion might induce the mobilization of EBMD cells but not EPC fractionation. We showed that TNF-*α* modulated the differentiation of bone marrow cells into *α*-SM actin positive cells in vitro. Thus, TNF-*α* might contribute to the activation, migration, and proliferation of SMCs in the injured artery, partly through inhibiting the mobilization of bone marrow cells.

We created models of vascular injury in which bone marrow cells from IL-6 KO mice were transplanted into WT mice (BMT mice) and then intimal hyperplasia and inflammation were immunohistochemically assessed after vascular injury.

Analysis by FACS showed that CD34^+^/Sca-1^+^ progenitor cells were more abundant in the peripheral blood of KO than WT mice at three days after, compared with before, injury when the two groups of mice did not significantly differ.

The numbers of apoptotic, possibly inflammatory cells in the neointima were much lower in the KO and BMT groups than in the WT group. Morphometric analysis at one week after injury showed smaller neointimal areas in the KO and BMT than in the WT group. Immunohistochemical assessment at one week revealed fewer CD34^+^ cells possibly containing EBMD cells in the KO and BMT groups than in the WT group. At week four no cells were CD34^+^ in the WT, KO, and BMT groups. Neointimal hyperplasia was significantly reduced in the KO and BMT groups compared with the WT group. Reendothelialization detected by staining with vWF antibody was more extensive in KO and BMT than in WT. Concentrations of VEGF and HGF in mouse sera determined using ELISA did not significantly differ among the groups before or at one week after vascular injury, indicating that vascular injury did not affect the concentrations of systemic growth factors in any of the three groups (submitted). The immunohistological findings were essentially similar between the BMT model and KO mice, meaning that IL-6 might signal a need for repair, mobilize endogenous bone marrow cells, and play an important role in the development of neointimal hyperplasia and reendothelialization after vascular injury. Some investigators have indicated that EBMD cells are unlikely to form a definitive smooth muscle cell lineage, instead of monocyte/macrophage lineages [37]. Our immunohistological findings also indicated that the neointimal portion after injury was negative for definitive SMC lineage markers. This finding indicated that the neointimal portion contained immature SM lineages that contributed to the increase in intima-media ratio and were mobilized by the inflammatory effects of IL-6. 

Moreover, our FACS data showed that CD34^+^/Sca-1^+^ progenitor cells might be involved in the reendothelialization detected by immunohistological staining, especially in the KO and BMT mice. These findings indicated that inflammatory cytokines signal a need for repair and that mobilized endogenous bone marrow cells play an important role in the process of neointimal hyperplasia after vascular injury. Inflammatory cytokines might contribute to the activation, migration, and proliferation of smooth muscle progenitor cells at sites of injury, through the mobilization of bone-marrow-derived cells (Figure 3(b)).

## 5. Future Perspectives on Therapy Using Bone-Marrow-Derived Cells to Prevent Restenosis

Percutaneous coronary intervention has become widely established as a treatment for atherosclerosis. However, PCI fails in a significant number of patients due to progressive vessel narrowing or post-PCI restenosis because PCI causes vessel wall injury and induces SMC proliferation with the subsequent abundant production of extracellular matrix. The endothelium is completely denuded by PCI and atherosclerotic lesions become mechanically dilated with tears in the luminal surface. Therefore, neointima formation in response to vascular injuries appears to be similar to the healing process. In addition to the conventional assumption that damaged tissues are repaired by individual parenchymal cells, accumulating evidence indicates that progenitors are mobilized to remote organs, where they differentiate into the required lineages and participate in organ repair and regeneration. Circulating bone-marrow-derived cells probably contribute substantially to vascular remodeling in humans with severely injured arteries. Thus, bone-marrow-derived cells might be an additional source of vascular cells that contribute to the repair and remodeling of vessel walls.

Although drug-eluting stents have significantly reduced rates of restenosis, delayed reendothelialization and subacute thrombosis persist. Endothelial progenitor cell captured by stents coated with antibody against CD34 represents a recent unique approach to EPC-mediated reendothelialization that has been adopted for clinical application [38, 39]. Moreover, recent advances in tissue engineering have led to the development of MSC-seeded stents [40]. Reendothelialization by bone-marrow-derived cells seems attractive and promising.

Treatments based on stem cell biology will become applicable to endovascular techniques beyond drug-eluting stents, but long-term outcomes will require careful observation.

## 6. Conclusion

We spatially and temporally investigated the cellular components involved in the development of neointimal hyperplasia in mice with vascular mechanical injury and bone-marrow transplantation. Bone-marrow-derived cells contributed to neointimal formation and transdifferentiated into immature SMCs or endothelium at injured vascular sites. The inhibition of inflammatory reactions via the paracrine effects of exogenous hMSCs might have helped to stabilize injured vascular regions through the mobilization of more endogenous bone-marrow-derived cells to the peripheral circulation. Granulocyte-colony stimulating factor mobilized more bone-marrow stem cells to the peripheral circulation, and these cells accumulated on the injured side of the vascular luminal. The inflammatory cytokines TNF-*α* and IL-6 mobilized endogenous bone marrow cells, which played an important role in the process of neointimal hyperplasia after vascular injury.

A mechanism for altering the transdifferentiation or paracrine capabilities of EBMD cells might be developed based on understanding of the distinct roles of various biological factors in the process of neointimal hyperplasia. We propose that these factors could become targets of treatment for patients with cardiovascular diseases. After the molecular mechanisms of the various factors involved in the capabilities of bone morrow-derived cells have been elucidated in vitro, promising targets to treat restenosis after PCI or atherosclerosis might be identified.
